# A Journey into Chaos: Creativity and the Unconscious**

**DOI:** 10.4103/0973-1229.77424

**Published:** 2011

**Authors:** Nancy C. Andreasen

**Affiliations:** * *Andrew H. Woods Chair of Psychiatry, W278GH, University of Iowa Carver College of Medicine, Iowa City, IA 52242, USA.*; ** *Revised and peer reviewed version of a Keynote Address for an International Seminar on Mind, Brain, and Consciousness, Thane College Campus, Thane, India, January 13-15, 2010*

**Keywords:** *Creativity*, *Complexity*, *Consciousness*, *Default mode*, *Functional imaging*, *Self-organising systems*, *The Unconscious*, *Resting state*, *REST*

## Abstract

The capacity to be creative, to produce new concepts, ideas, inventions, objects or art, is perhaps the most important attribute of the human brain. We know very little, however, about the nature of creativity or its neural basis. Some important questions include how should we define creativity? How is it related (or unrelated) to high intelligence? What psychological processes or environmental circumstance cause creative insights to occur? How is it related to conscious and unconscious processes? What is happening at the neural level during moments of creativity? How is it related to health or illness, and especially mental illness? This paper will review introspective accounts from highly creative individuals. These accounts suggest that unconscious processes play an important role in achieving creative insights. Neuroimaging studies of the brain during “REST” (random episodic silent thought, also referred to as the default state) suggest that the association cortices are the primary areas that are active during this state and that the brain is spontaneously reorganising and acting as a self-organising system. Neuroimaging studies also suggest that highly creative individuals have more intense activity in association cortices when performing tasks that challenge them to “make associations.” Studies of creative individuals also indicate that they have a higher rate of mental illness than a noncreative comparison group, as well as a higher rate of both creativity and mental illness in their first-degree relatives. This raises interesting questions about the relationship between the nature of the unconscious, the unconscious and the predisposition to both creativity and mental illness.

## Introduction

Creativity is one of our most valued human traits. It has given human beings the ability to change the world that they live in; and it has also, paradoxically, given them the ability to adapt to changes in the world over which they have no control. Our highly developed capacity to develop and implement new ideas arises from our highly developed human brain. Understanding how creative ideas arise from the brain is one of the most fascinating challenges of contemporary neuroscience.

## What is Creativity?

The first step in studying creativity is to define what it is. One of the first issues that must be addressed is the relationship between creativity and high intelligence. Since the same word, genius, is often used to refer to people who are highly creative and also to people who are highly intelligent, a common assumption is that creativity and high intelligence are the same thing. This is, however, a misconception.

Perhaps, the strongest demonstration of the difference between these two mental capacities comes from the work of Lewis Terman (1925–1959). Beginning in 1921, Terman conducted a landmark longitudinal study of children born in California in 1910 and after, who had IQs in the 135 to 200 range. He evaluated them at regular intervals for the next few decades, and the study was continued by his successors so that they were followed into middle and late middle age. These individuals, who came to be known as the “Termites,” were thoroughly studied; variables included information about general health, mental health, age at puberty, height and weight, social skills, educational achievement, marriage and divorce, occupation, public recognition and many others. Contrary to popular beliefs at the time, the Termites were not undersized, socially inept or badly adjusted. Instead, they enjoyed better general and mental health than their comparison group, and they were also more successful in their later lives and careers. Somewhat surprisingly, however, they were not found to have made highly creative contributions after reaching adulthood. When the cohort of 757 individuals available for follow-up at mid-life were evaluated, only three were engaged in creative activities (one Oscar-winning film director and two successful writers). Additional confirmation that creativity and high intelligence are different mental capacities comes from approaching the question from a different direction: the study of highly creative people who have been IQ-tested. Several studies have shown that groups of highly creative people (e.g., well-known writers, successful architects) have mean IQs in the 120 range (Andreasen, 1987; Mackinnon, 1965). An IQ in this range is considered to be “superior,” but it is not in the “genius” range.

If creativity is not equivalent to a high IQ, then how else might it be defined and measured? Several different approaches have been taken to address this question. One has been to develop tests specifically designed to measure creativity and to designate people who achieve high scores on these tests as creative. The basic assumption behind most such tests is that creativity can be defined as having a capacity for achieving a high level of *divergent thinking*. Divergent thinking is defined as the ability to come up with a large number of responses to an open-ended probe; it is contrasted with *convergent thinking*, which tends to apply a sequential series of steps to answer a question that has only one possible solution (Runco and Marz, 1992). An example of a probe used to assess divergent thinking is asking: *How many uses can you think of for a brick?* A series of similar questions can be asked and then used to create a score that is a continuous measurement of divergent thinking (Torrance, 1998). This approach is favoured by some psychologists as a way of achieving an objective measure of creativity.

An alternative approach is to define creativity operationally. That is, people who have produced some type of creative output are designated as creative based on their achievements. When this method is used, it is typically in conjunction with an approach known as the “case study method.” People are selected because they have achieved a high level of success and recognition in fields such as architecture, writing, mathematics and physics. Often a specific criterion of success is used, such as having won a major prize or award (e.g., Fields Medal, Nobel Prize, Pulitzer Prize, Lasker Award). These people are further assessed using structured interviews about their work habits and thought processes, personality tests and measures of cognition. The commonalities that they share are considered to be characteristics of creative people and their cognitive style. An important recent spin-off of this approach is to conduct neuroimaging studies of such people in order to examine the neural basis of creativity.

## The Interface Between Creativity and Unconscious Processes

One entry into understanding the neural basis of creativity is to listen to people as they describe how their ideas come to them. The most famous example is perhaps Archimedes, who was confronted with the challenge of determining whether an irregularly shaped golden crown was made of pure gold or an alloy. The solution came to him in a flash of insight as he got into a bathtub, sat down and suddenly realised that he could measure its density by measuring the amount of water it displaced divided by its weight, just as water was displaced by his body in the tub. As legend has it, he shouted “Eureka!” (Greek for “I have found it!”) and ran out of the house naked because he was so excited that he forgot to dress.

This archetypal story has been echoed over and over by creative individuals as they describe how they get their ideas and inspiration. The creative process moves through stages. It begins with *preparation*, a time when the basic information or skills are assembled. It continues on to *incubation*, a relaxed time during which the person does not work consciously to solve the problem, but when connections are unconsciously being made. This then leads eventually to *inspiration*, the eureka experience when the person suddenly sees the solution. It ends with *production*, a time when the insights are put into a useful form. The specifics of this basic process will vary depending on the type of creativity; writing a novel is different from identifying a new chemical synthesis. But the basic process and principles are the same across many different types of creativity. Describing their subjective experiences, creative people say the same things repeatedly:

“I can’t force inspiration. Ideas just come to me when I’m not seeking them-when I’m swimming or running or standing in the shower.” “It happens like magic.” “I can just see things that other people can’t, and I don’t know why.” “The muse just sits on my shoulder.” “If I concentrate on finding the answer it never comes, but if I let my mind just wander, the answer pops in.” (Andreasen, 2005.)

Here, for example, is Poincare’s description of how he discovered Fuchsian functions:

One evening, contrary to my custom, I drank black coffee and could not sleep. Ideas rose in crowds; I felt them collide until pairs interlocked, so to speak, making a stable combination. By the next morning I had established the existence of a class of Fuchsian functions, those which come out from the hypergeometric series; I had only to write out the results, which took but a few hours. (Poincare, 2001, p 220.)

If we try to understand these descriptions using the framework provided by our understanding of the mind and brain, then we are led to the conclusion that the creative process arises from the unconsciousness rather than occurring as a conscious process. The person is typically in some type of reverie or dissociative state when the mind wanders freely and thoughts and images float around without censorship. During this fluid time, the brain is probably working feverishly, despite the subjective sense of reverie and relaxation. As Poincare says, “ideas rose in crowds.” At the neural level, it is as if the association cortices are working actively, throwing out feelers for possible connections between unrelated capacities-verbal and visual spatial associations, abstract and concrete associations, colours, images, concepts…a veritable primordial soup of thought. Then, within this primordial soup, ideas “collide until pairs interlock…making a stable combination.” As we think about this process using the terminology of mind and brain, then the primordial soup is the unconsciousness and process of making connections must arise from the efforts of the association cortex.

## Random Episodic Silent Thought and the Default Mode Network: Visualising the Unconsciousness with Functional Imaging Techniques

Although the subjective state of allowing the mind to wander freely has been recognised as a discrete mental activity for many years, few tools have been available to understand how these thoughts actually arise in the mind or brain. The development of functional imaging technologies has changed that. Using the tools of positron emission tomography (PET) and functional magnetic resonance (fMR) imaging, we are now able to visualise and measure *how the brain thinks*.

Our group in fact conducted the first empirical examination of intrinsic neural activity during “free association,” as inferred from regional cerebral blood flow during the early era of PET research (Andreasen *et al*., 1995). During this early era of functional imaging research, the basic study design involved a comparison between two tasks. One, the experimental task, was the cognitive ability being studied-verbal fluency, remembering lists of words, recognising faces or focusing attention. The experimental task was usually compared with a neutral or “baseline” task; frequently, this baseline task was the “resting state,” during which subjects were instructed to relax or rest. In essence, they were being given the same instructions as often occurred during free association: relax and simply think about whatever comes into your mind.

The expectation that the “resting state” would be a neutral or quiet activity seemed ludicrous to our imaging research group. Arguing that the brain never “rests,” we used PET to examine which brain regions were more active during the “resting state” in healthy normal volunteers, a condition during which the subjects were allowed to let their minds wander freely (Andreasen, 1995). In other words, we treated the “resting state” as an experimental condition in its own right. When we did this, the results were not surprising. We found activations in multiple regions of association cortex, including frontal, temporal and parietal, as well as the retrosplenial cingulate. Essentially, we demonstrated that the process of “free association” allows the association cortices of the human brain to converse with one another in a free and uncensored manner! When we systematically debriefed the subjects about their mental activity during this condition, we learned that they were engaged in random free-floating self-referential thoughts about the past, present and future, what is conventionally termed “episodic memory” (as contrasted with “semantic memory”) in the field of cognitive science, or free association in the field of psychoanalysis. Therefore, we suggested with a touch of irony that the “resting state” should be referred to as random episodic silent thought, for which REST is an appropriate acronym. We were not visualising a passive silent brain during the “resting state,” but rather a brain that was actively connecting thoughts and experiences.

This observation lay relatively dormant for a number of years, but the study of REST has now emerged as one of the “hot topics” of contemporary cognitive neuroscience. Its study has been facilitated by the increasing use of fMR, a functional imaging technique that is more widely available and less invasive than PET because it requires no radiation exposure. In this more recent literature, REST has been renamed the default mode, and its associated network is now referred to as the default mode network (Buckner *et al*., 2008; Raichle and Snyder, 2007). The coherent low frequency fluctuations in fMR BOLD activity are now thought to be an inherent property of the human brain and to reflect intrinsic connectivity networks.

## A Journey into Chaos: How do Ideas Arise from the Unconscious?

Recognising that the association cortices are active during unconscious thought is a beginning, but it does not tell us how the connections themselves are made. It tells us the “where,” but not the “how.” What process occurs when ideas arise in crowds and then collide “until pairs interlock”? How does a random process eventually lead to something meaningful? *How does the brain think*? This is one of the deepest questions in modern neuroscience.

An outdated answer would be that the prefrontal cortex acts as the executive that supervises the process. This answer is not adequate, however, because it is based on an outdated localisation model that imputes differing responsibilities to different brain regions. More modern and current models of the brain conceptualise it as comprised of distributed circuits comprised of nodes that mutually share the responsibility for creating its outputs.

But who decides what the outputs will be, if there is no executive? To answer this question, we must turn to the concepts of self-organising systems and chaos theory. Chaos theory, also known as complexity theory, is the study of dynamic and nonlinear processes and of self-organising systems (Gleick, 1987). Self-organising systems can be seen all around us, once we begin to look for them. We see them in the flocking of birds, the schooling of fish and the changing global ecosystem. All these things produce a form of organisation in which the control is not centralised, but rather is distributed throughout the entire system. The system is dynamic, and changes arise spontaneously and frequently produce something new. Seen within this context, the human brain is the ultimate self-organising system, and creativity is one of its most important emergent properties.

## Genius and Insanity: The First Iowa Study of Creative Genius

An initial study of highly creative individuals was conducted at the University of Iowa during the 1970s and 1980s (Andreasen, 1987). This study was facilitated by the fact that a pool of creative people was readily available locally; the University of Iowa is the home of the Writers’ Workshop, the oldest and most famous creative writing programme in the United States, and perhaps in the world. Founded in 1936, it has been home to many of America’s most distinguished writers at some point in their careers. Among them: Kurt Vonnegut, John Irving, Robert Lowell, Phillip Roth, John Cheever, Flannery O’Connor and many more. Tennessee Williams was also a student in the Iowa Drama Workshop. Because I was a faculty member in the English Department before changing careers to study medicine, I knew many workshop writers. When I decided to conduct a study of the relationship between creativity and mental illness during the 1970s, the study was relatively easy to conduct because of the rich trove of available subjects.

The working hypothesis behind this study was that there was a relationship between creativity and psychosis, particularly schizophrenia. The empirical evidence driving this hypothesis consisted of several famous cases. James Joyce had a daughter with schizophrenia and had many schizotypal traits. Albert Einstein had a son with schizophrenia and was also somewhat schizotypal and eccentric. Bertrand Russell had many family members with schizophrenia or psychosis: his aunt, uncle, son and grand-daughter. There were also good theoretical reasons for expecting an association between creativity and schizophrenia. Psychotic individuals often display a capacity to see the world in a novel and original way, literally, to see things that others cannot. Might not the cognitive traits possessed by people with psychosis have something in common with those possessed by creative people, who also can sometimes see things that others cannot?

Although the hypothesis had a good empirical and theoretical basis, it was not confirmed in this early study. Instead, the writers had a high rate of mood disorder. Furthermore, their first-degree relatives also had a high rate of mood disorder, as compared with an educationally and IQ-matched control group. Why was there such a mismatch between hypothesis and results? One possibility is that the hypothesis was simply wrong. Another, however, is that limiting the sample to novelists and poets may have biased the results. Two of the three people who influenced the initial hypothesis, Einstein and Russell, were scientists…and scientists who inhabited a world shaped by the arcane abstractions of mathematics. Would the findings have been different, if scientists had been studied instead of artists?

## Adding Neuroimaging and ‘Seeing’ the Brain: The Second Iowa Study of Creative Genius

These thoughts percolated for several decades, and more questions were added as well. The tools of structural and functional neuroimaging became available during the 1980s and 1990s, offering a window into understanding creativity that opened vistas unreachable through introspection and structured interviews. What would we find if we studied the brains of highly creative people and compared them with a noncreative comparison group? Would they differ in brain structure? In functional activity? Would artists and scientists differ in their functional brain activations or in their brain structure? The second Iowa Study of Creative Genius was finally initiated in order to address these questions.

The design of the study is a classic case-control comparison. When it is completed, the subjects will include 30 highly creative artists, 30 highly creative scientists and 30 noncreative comparison subjects. The definition of “highly creative” is operational. Individuals are recruited for the study if they have won a major award in their field–Fields Medal for mathematics; Nobel prizes for chemistry, physics, physiology or medicine; Pulitzer Prizes or National Book Awards; National Medal of Science and other similar high levels of recognition for creative achievement.

The approach involves the “intensive case study” method. Individuals come to Iowa City, where they spend two days participating in interviews and tests. A special structured interview is used to evaluate their family history, early life and personal history, their history of creative accomplishments, their work habits, the ways they develop their ideas and complete their work and their personal and family history of mental illness. They are evaluated with a WAIS-III (conceptualised as a way to assess different facets of intellectual ability rather than as a test of intelligence) and a Temperament and Character Inventory (as an evaluation of multiple facets of personality). They are also evaluated with 3T structural and fMR scans.

The selection of a design for the fMR studies was challenging. As described above, the core component of the creative process is usually a flash of insight that leads to a new idea or the solution of some problem. It cannot be forced. Furthermore, the very nature of fMR study design runs counter to the nature of the creative process. Because fMR has a very poor signal to noise ratio, tasks must be performed repeatedly in order to extract a signal. The most powerful way to deal with the poor signal to noise ratio is to use an on-off block design, during which an experimental task (“on”) is alternated with a control task (“off”); depending on the task, the on-off repetitions occur 7 to 10 times in a run, and the run is repeated two to three times. How can a creative person be expected to come up with a “creative idea” when lying in an MR scanner and being confronted with such a repetitive and tedious series of tasks?

The challenge of designing a suitable group of fMR tasks was resolved by finally deciding that expecting subjects to repeatedly come up with “creative thoughts” was impossible. On the other hand, conceptualising the tasks in terms of assessing brain networks involved in creativity was highly feasible.

As described above, the brain regions most likely to be involved in the creative process are the association cortices, those brain regions that are most active during REST when a person is engaged in free-ranging and uncensored thought. Therefore, a simple and logical solution to the challenge of designing a functional imaging study of creativity was to select mental tasks that would engage the association cortices. Consequently, two tasks are used in the study to tap into the activity of association cortex. One is a word association test, during which the subject silently reads a word and then responds with the first word that comes to mind. The other is a picture association test, during which the subject looks at a picture and responds with the first thought about the picture that comes to mind. These two tasks tap into the process of making verbal and visual associations. A third task was selected in order to examine brain activity during abstract pattern recognition, a process similar to that occurring during some aspects of scientific creativity. This task is based on the Raven Progressive Matrices. Finally, we also collect two sessions of REST, in order to examine activity in the default state network.

**Figure 1:**
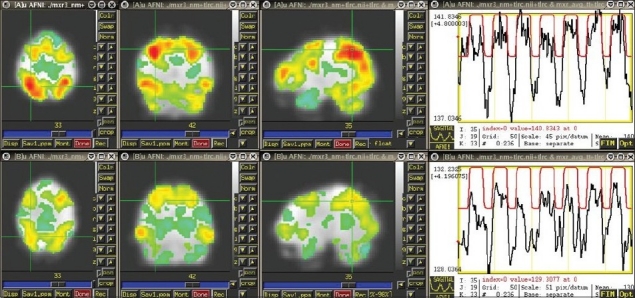
A creative subject (above) and a control subject (below) during an fMR task using a boxcar design.

This study is still in its early stages, because recruitment is challenging, funding is limited and the study of each subject is very time-consuming. However, some conclusions are already beginning to emerge. *First*, it is now clear that the choice of tasks and the implementation of the block design for the fMR component of the study were well reasoned and well implemented. The tasks all produce robust activations in plausible regions. During word association, activations occur in left and right middle and inferior frontal regions, anterior cingulate and left middle temporal gyrus [Figure 1]; these are the association cortex regions used for language. During picture association, activations occur in primary visual cortex, bilateral fusiform gyri, left and right angular gyri and bilateral middle and inferior frontal gyri; these are regions used for making visual associations. During pattern detection, activations occur in bilateral fusiform gyri, anterior cingulate, bilateral precuneus, bilateral superior parietal lobes and bilateral insula; these are regions used for visual-spatial perception. *Second*, it is also clear that the creative individuals have stronger activations in these regions than do the control subjects. And *third*, the activations are quite similar in artists and scientists, suggesting that the brain may know no dichotomy between these two disciplines. Sample size is still too small to make inferences about different patterns of mental illness in the two groups.

## Concluding Remarks [see also Figure 2]

**Figure 2:**
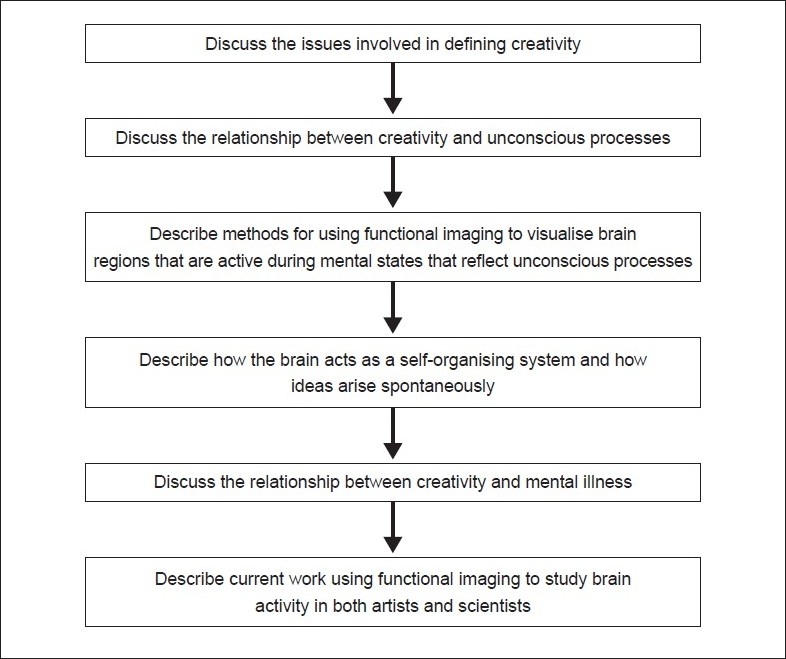
Flow chart of paper

Consciousness, the unconscious and creativity are all important facets of the human mind. They are extremely difficult to study rigorously. This paper has described several approaches that can be used. In particular, functional imaging studies offer an objective tool for studying these complex facets of the human mind.

### Take home message

The creative process is characterised by flashes of insight that arise from unconscious reservoirs of the mind and brain. Imaging studies indicate that these reservoirs reside in association cortices. During the creative process, the brain works as a self-organising system.

## Questions That This Paper Raises

1. Are there structural brain differences between creative and noncreative people?
2. What are the personality traits that characterise creative people?
3. Is the creative process for performing artists similar to that described for other artists and for scientists?
4. What types of functional imaging designs lend themselves to the study of creativity?
5. Could the methods for studying artists and scientists also be adapted to the study of other domains that are less obviously “creative,” such as business or politics?

## About the Author

*Nancy C. Andreasen, M.D., Ph.D., is Andrew H. Woods Chair of Psychiatry and Director of the Psychiatric Iowa Neuroimaging Consortium at The University of Iowa Carver College of Medicine. She is actively involved in neuroimaging research, which involves the use of structural MR imaging, functional MR and positron emission tomography. In addition, she leads a team working on threedimensional image analysis techniques, to integrate multi-modality imaging and to develop innovative methods for analysing structural and functional imaging techniques in an automated manner. She conducts several investigator-initiated projects in the area. She has also contributed to nosology and phenomenology by serving on both the DSM and DSM IV Task Forces and developing the first widely used scales for rating the positive and negative symptoms of schizophrenia. She is past president of the American Psychopathological Association and the Psychiatric Research Society. She is a member of the Institute of Medicine of the National Academy of Science and was elected to serve on its governing council for two four-year terms. She is also a fellow of the American Academy of Arts and Sciences and of the Society for Neuroscience. She has won the President’s National Medal of Science for 2000. She has also received many other awards, including the Interbrew-Baillet-Latour Prize from the Belgian government, the Rhoda and Bernard Sarnat Prize from the Institute of Medicine, the Lieber Prize for Outstanding Schizophrenia Research, the Sigmund Freud Award from the American College of Psychoanalysis, the Kolb Award and the Sachar Award from Columbia University. The American Psychiatric Association has awarded her its Prize for Research, its Kempf Award for Mentoring, the Hibbs Award, its Adolph Meyer Award and its Distinguished Service Award. She has received the Stanley Dean Award from The American College of Psychiatrists, as well as its Distinguished Service Award. Most recently, Dr. Andreasen won the Vanderbilt Prize for Biological Science. She was Editor-in-Chief of The American Journal of Psychiatry for 13 years, completing her third term in 2005. She has written two widely praised books for the general public, “The Broken Brain: The Biological Revolution in Psychiatry” (1983) and “Brave New Brain: Conquering Mental Illness in the Era of the Genome” (2001). More recently, she has written “The Creating Brain: The Neuroscience of Genius” (2005). She has also authored, co-authored or edited twelve other scholarly books and over 500 articles. She heads the Honorary International Editorial Advisory Board of the Mens Sana Monographs*
